# Is the Climate Right for Pleistocene Rewilding? Using Species Distribution Models to Extrapolate Climatic Suitability for Mammals across Continents

**DOI:** 10.1371/journal.pone.0012899

**Published:** 2010-09-22

**Authors:** Orien M. W. Richmond, Jay P. McEntee, Robert J. Hijmans, Justin S. Brashares

**Affiliations:** 1 Department of Environmental Science, Policy & Management, University of California, Berkeley, California, United States of America; 2 Museum of Vertebrate Zoology, University of California, Berkeley, California, United States of America; 3 Department of Environmental Science and Policy, University of California Davis, Davis, California, United States of America; 4 Department of Environmental Science, Policy & Management, University of California, Berkeley, California, United States of America; University of California, Berkeley, United States of America

## Abstract

Species distribution models (SDMs) are increasingly used for extrapolation, or predicting suitable regions for species under new geographic or temporal scenarios. However, SDM predictions may be prone to errors if species are not at equilibrium with climatic conditions in the current range and if training samples are not representative. Here the controversial “Pleistocene rewilding” proposal was used as a novel example to address some of the challenges of extrapolating modeled species-climate relationships outside of current ranges. Climatic suitability for three proposed proxy species (Asian elephant, African cheetah and African lion) was extrapolated to the American southwest and Great Plains using Maxent, a machine-learning species distribution model. Similar models were fit for *Oryx gazella*, a species native to Africa that has naturalized in North America, to test model predictions. To overcome biases introduced by contracted modern ranges and limited occurrence data, random pseudo-presence points generated from modern and historical ranges were used for model training. For all species except the oryx, models of climatic suitability fit to training data from historical ranges produced larger areas of predicted suitability in North America than models fit to training data from modern ranges. Four naturalized oryx populations in the American southwest were correctly predicted with a generous model threshold, but none of these locations were predicted with a more stringent threshold. In general, the northern Great Plains had low climatic suitability for all focal species and scenarios considered, while portions of the southern Great Plains and American southwest had low to intermediate suitability for some species in some scenarios. The results suggest that the use of historical, in addition to modern, range information and randomly sampled pseudo-presence points may improve model accuracy. This has implications for modeling range shifts of organisms in response to climate change.

## Introduction

Species distribution models (SDMs), also known as bioclimatic or ecological niche models, climate envelope models and predictive habitat distribution models, statistically relate known species occurrences with environmental variables in order to predict potential regions of suitability for species or communities [1], [2]. There are two common uses of SDMs: (1) interpolation, or predicting entire distributions of organisms from limited occurrence data within the existing range and (2) extrapolation, or predicting suitable regions for species under novel geographic or temporal scenarios. SDMs are the most common tool used for predicting the potential ranges of organisms, and they are increasingly being employed to address biodiversity conservation, especially in the context of climate change [3]. For example, SDMs are used to identify areas outside known ranges that might support important taxa [4], evaluate sites for reserve selection [5], [6], prioritize areas for reintroductions [7], predict the potential range and rate of spread of invasives [8]–[11] and predict the responses of existing species' ranges to climate change [12], [13]. An important emerging application of SDMs is the prediction of potential ranges of organisms undergoing “assisted migration,” or deliberate introduction to areas outside of the present (and perhaps historical) range, in order to more proactively manage rare or threatened species in the face of climate change, habitat loss and other pressures [14]–[17].

Despite the growing use of SDMs for extrapolation, substantial uncertainties remain about the accuracy of model predictions when transferred in space or time [18]. Sources of error in SDM projections stem from violations of four key model assumptions (Table 1). First, a species is assumed to be at equilibrium with climatic conditions in the current range; i.e., a species is present in almost all regions of the training area where climatic conditions are suitable. However, biotic interactions can preclude a species from occurring in climatically suitable regions (e.g., widespread range contractions in mammals and birds linked to human disturbance [19]) and dispersal limitation can prevent a species from encountering suitable areas [20], resulting in biased training data. Second, it is assumed that the climatic niche is stable, such that climatic factors that limit a species' occurrence in the current range will also be limiting in the extrapolated area [21]. In order for this to be true, it is assumed that new ecological relationships (e.g., competition, predation) and new behavioral and/or evolutionary adaptations in the introduced area are negligible. While new evolutionary adaptations are less likely to occur on short timescales, unpredictable biotic interactions often lead to ecological surprises [22] and behavioral adaptations, such as migration, may occur quite rapidly [23]. Third, it is assumed that training samples are representative of the environmental conditions across the current range. In reality, species records used for model training, usually consisting of localities from museum collections, are often spatially clumped or incomplete and therefore not representative of the full range of environmental conditions in the current range [24]–[27]. Fourth, it is assumed that climatic conditions in the current and extrapolated areas are analogous. However, novel climatic conditions may exist in the extrapolated area [22] and SDMs may inappropriately extrapolate beyond the range of values for environmental predictors found in the native range [28]. Violations of assumptions 1 and 3 are expected to lead to increased errors of omission (false negatives) in model predictions, while violations of 2 and 4 can lead to both errors of omission or commission (false positives; Table 1). Several studies have reported high errors of omission when SDMs are used for extrapolation [29]–[34], suggesting that addressing these violations of model assumptions, particularly those that cause errors of omission, could improve model performance. Performance is additionally influenced by model [35], variable [36] and threshold [37], [38] selection, among other factors.

**Table 1:** Four assumptions made in using species distribution models (SDMs) to extrapolate climatic suitability to new regions, how these assumptions are violated, the consequences of violations for model performance and solutions to improve model performance.

SDM assumptions	Violations of SDM assumptions	Consequences of violating SDM assumptions on model performance	Solutions to improve SDM performance
Assumption 1: Species is at equilibrium with environmental conditions in its native range	Native range is restricted by biotic interactions (e.g., competition, predation, human disturbance, etc.)	Underprediction of potential regions of suitability	Use historical range information for model training
	Native range is restricted by dispersal limitation	Underprediction of potential regions of suitability	
Assumption 2: Niche stability	Evolutionary or behavioral adaptation to environmental conditions in introduced area	Underprediction of potential regions of suitability	Shorten timescale of analysis
	New ecological relationships in introduced range	Overprediction or underprediction of potential regions of suitability	
Assumption 3: Training samples are representative of environmental conditions in native range	Training samples are biased	Underprediction of potential regions of suitability	Use design- or model-based environmental stratifications to target underrepresented areas for additional field data collection
			Generate random pseudo-presence points across native range
	Few training samples are available	Underprediction of potential regions of suitability	Generate adequate number of random pseudo-presence points from native range
Assumption 4: Climatic conditions between training and introduced areas are analogous	Novel climatic conditions occur in introduced area; modeled responses extrapolate beyond range of values for environmental predictors found in native range	Overprediction or underprediction of potential regions of suitability	Use a clamping procedure to limit predictions in regions with novel climatic conditions

Here we use the controversial “Pleistocene rewilding” proposal [39], [40] as a novel example to address some of the challenges of extrapolating modeled relationships outside native ranges. The proposal calls for introducing close extant relatives or ecological surrogates of megafauna that went extinct at the end of the Pleistocene to North America to restore lost ecological and evolutionary processes, while simultaneously conserving species currently threatened with extinction on other continents [40]. However, most of the proposed proxy species originate from tropical and sub-tropical Africa and Asia, thus North America's colder temperatures and greater seasonality may preclude establishment. Here we assess the projected climatic suitability of proposed North American introduction areas, the American southwest and Great Plains, for four focal species from Africa and Asia (Table 2) using pseudo-presence training data from modern vs. historical native ranges and Maxent [41], a maximum entropy model. Three of our focal species, the Asian elephant (*Elephas maximus*), African cheetah (*Acinonyx jubatus*; hereafter “cheetah”) and African lion (*Panthera leo*; hereafter “lion”), were among the 11 candidate species in the Pleistocene rewilding plan [40]. For evaluation purposes, we included a fourth species not included in the rewilding proposal, the Gemsbok (*Oryx gazella*; hereafter “oryx”), because it is an Old World mammal that was introduced to New Mexico, U.S.A., in 1969 and has since naturalized [42]. The primary aims of the study were to: (1) model climatic suitability for each focal species in modern and historical native ranges, assess model performance and identify the climate variables that made the largest contributions to modeled responses; (2) assess the sensitivity and accuracy of model outputs in the native range to training points generated from modern vs. historical ranges, training point variation and different thresholds applied to the model outputs; (3) extrapolate models trained on native modern and historical ranges to North America and evaluate the concordance between predicted climatic suitability and the proposed introduction regions from the Pleistocene rewilding plan; and (4) use known localities where oryx have established in Texas and New Mexico to provide an independent test of the modeling procedure.

**Table 2:** Focal species examined in the study.

Common Name:	Scientific Name:	Continent of Origin:	Pleistocene rewilding proxy for:
Asian elephant	*Elephas maximus*	Asia	Mastodon, mammoth, gomphotheres
African cheetah	*Acinonyx jubatus*	Africa	American cheetah
African lion	*Panthera leo*	Africa	American lion
Oryx or gemsbok	*Oryx gazella*	Africa	n.a.

## Methods

### Modeling Approach

The main goal of our modeling approach was to address two violations of model assumptions and thereby improve model performance. First, to address violation of the assumption of equilibrium due to human-caused range contractions (Assumption 1, Table 1), we compared projected areas of climatic suitability using training data from modern vs. historical ranges. Historical range sampling allowed us to include training information from areas that are presently unoccupied yet climatically suitable. Including historical range information has improved model performance in other cases [43], but the coarse resolution inherent to historical range maps also has the potential to introduce bias. A recent study used pooled modern and historical locality information to improve model performance when extrapolating to a new geographic area [31]. However, in our case it was unnecessary to pool modern and historical data from the native range, because for each focal species the historical range was larger and encompassed all of the modern range. It was also not feasible to pool the native and North American locality data for the oryx because its introduced range was so restricted.

Second, to address violation of the assumption of representative training samples (Assumption 3, Table 1) we used “pseudo-presence data”—points randomly sampled from across the range of the focal species—rather than actual occurrence localities from museums, herbaria, or field surveys. While using design- or model-based environmental stratifications to target underrepresented areas for additional field data collection has been suggested to address the incomplete sampling problem [44], conducting fieldwork is costly across large geographic areas. In contrast, random pseudo-presence data can easily be generated using Geographical Information System (GIS) software. Using pseudo-presence data may lead to overpredictions in characterizing climatic suitability because large-scale “extent-of-occurrence” geographical ranges include some unsuitable areas and thus tend to exaggerate actual occurrence [45]–[47]. However, a recent study concluded that some SDMs, including Maxent, are to some degree robust to locational errors in occurrence data [48]. Since different sets of randomly sampled training points should produce different model outcomes, we assessed the effect of training point variability on model performance.

While not the focus of this study, we also attempted to minimize violations of the other two model assumptions (Assumptions 2 and 4, Table 1). Since we were assessing short-term climatic suitability relevant to the scale of a proposed species introduction program, we assumed niche stability (i.e., negligible effect of new ecological interactions and evolutionary or behavioral adaptation of focal species to climatic conditions in the extrapolated range). Climatic conditions in native vs. projected ranges (Africa and Asia vs. North America, respectively) were not completely analogous, potentially violating Assumption 4 (Table 1). However, Maxent implements a procedure called “clamping” (see Modeling Procedure) that prevents modeled responses from being extrapolated beyond the range of values for environmental predictors found in native range. We did not systematically address the problem of novel climatic conditions, but this issue can be approached by examining the edges of species' climate envelopes [22].

### Species Input Data

The model training data consisted of random pseudo-presence points that were generated within the modern and historical geographical distributions of the Asian elephant, cheetah, lion and oryx. We did not distinguish between subspecies or races of the focal species, but rather modeled each species as a single group. Thus, for the oryx we lumped the three subspecies *Oryx gazella gazella*, *Oryx gazella beisa* and *Oryx gazella calliotis* into a single group for modeling. Note that an alternative classification system has the Gemsbok as one species (*Oryx gazella*), and the East African Oryx as another (*Oryx beisa*) with two subspecies of its own, the East African Oryx “proper” (*Oryx beisa beisa*) and the Fringe-eared Oryx (*Oryx beisa calliotis*) [49]. To ensure that the full range of climatic conditions was sampled from each species' distribution, we examined the relationship between the number of points used in model training and predictive performance and selected 100 points for subsequent model fitting (Text S1, Figure S1). Thus, for each species and time period we generated ten sets of 100 random pseudo-presence training points within the range using Hawth's Tools [50] in ArcMap 9.3.1 [51]. We obtained modern range maps from the highest-resolution sources available at the time of the analysis for the Asian elephant [52], cheetah [53], lion [54] and oryx [55], [56]. The dates of historical range maps varied by species. The oldest localities included in the historical range data for the Asian elephant dated from approximately 1700 BC [57], for the cheetah from 0 AD [58] and for the lion from 480 BC [59]. The time period for the oryx's historical range data was unreported but is estimated to be no more than a few hundred years [60], [61].

### Climate Input Data

We used climate data from WorldClim, ver. 1.4 (http://www.worldclim.org/), a set of global climate layers that were generated through interpolation of average monthly climate data from weather stations tabulated from 1950–2000 [62]. We utilized climate grids that were aggregated to a resolution of 2.5 minutes. For all species we used ten bioclimatic variables as predictors: MTEMP  =  annual mean temperature; TEMPR  =  mean monthly temperature range; ISO  =  isothermality (mean monthly temperature range/temperature annual range); TEMPS  =  temperature seasonality (standard deviation of monthly temperature); MTWM  =  maximum temperature of the warmest month; MTCM  =  minimum temperature of the coldest month; PREC  =  annual precipitation; PRECS  =  precipitation seasonality (coefficient of variation of monthly precipitation); PWQ  =  precipitation of the wettest quarter; and PDQ  =  precipitation of the driest quarter [62]. Further information about the extent of the climate grids used in the modeling can be found in Text S2.

The WorldClim climate data were temporally matched with modern ranges but not with all parts of historical ranges; e.g., for the Asian elephant the oldest part of the historical range dated from 3,700 cal yr B.P. The Holocene (approximately the last 11,500 years) lacked large Northern Hemisphere ice sheets and is generally characterized as a warm and stable period with some episodes of apparent rapid climate change, particularly during the mid-Holocene extending from 7,000–5,000 cal yr B.P. [63], [64]. By about 4,000 cal yr B.P., Earth's climate had become fairly similar to today's [65]–[67], thus we made the simplifying assumption that pseudo-presence data sampled from historical ranges could be adequately modeled using climate data from the latter half of the twentieth century, especially when assessed in combination with the results of the models trained on present-day ranges.

### Modeling Procedure

We modeled climatic suitability for each focal species in its native range and made predictions of climatic suitability in North America using maximum entropy species distribution modeling (Maxent ver. 3.3.0), a general-purpose machine learning method [41], [68]. Recent studies compared the performance of several SDMs and Maxent outperformed many of the other methods [9], [35], [69]–[71]. The Maxent model generation approach requires only presence data (not absence data), can utilize both continuous and categorical data, can incorporate interactions between different variables and yields continuous outputs, allowing fine distinctions to be made between the modeled suitability of different areas. Starting with a set of samples from a distribution over some defined space (species locations), as well as a set of features on this space (environmental variables), Maxent estimates the target distribution of predicted climatic suitability by finding the distribution of maximum entropy, or closest to uniform, subject to the constraint that the expected value of each feature under this estimated distribution matches its empirical average [41]. This is equivalent to finding the maximum likelihood Gibbs distribution. Further discussion of Maxent and our application of the model, specifically issues of regularization multipliers, feature types and clamping, can be found in Text S3, Text S4 and Figure S2. The software and complete information for this method are available from http://www.cs.princeton.edu/~schapire/maxent, or see Phillips et al. (2006).

### Model Evaluation and Thresholding

We separately generated pseudo-presence testing data in Maxent to evaluate model outputs in native ranges (100 pseudo-presence points per run per species per time period). Maxent outputs the area under the Receiver Operating Characteristic curve (AUC), a threshold-independent measure, as one measure of model performance. AUC values range from 0 to 1 and measure the ability of a model to discriminate between sites where a species is present and sites where it is absent [72], [73]. A score of 1 indicates perfect discrimination while a score of 0.5 indicates discrimination that is no better than a random guess. AUC is widely used to evaluate SDM outputs, although its use has come under some criticism [74]. AUC scores allowed us to assess how well the modeled climatic suitability matched testing pseudo-presence points from native modern and historical ranges, but could not be used to evaluate projected climatic suitability in North America. The spatial extent of the naturalized North American oryx population was too small to obtain enough independent samples to statistically test the projected North American oryx distribution. Thus, we evaluated the accuracy of the projected oryx distribution qualitatively by examining how well Maxent's predictions of suitability overlapped known localities where oryx have naturalized in New Mexico and Texas.

It is often desirable to convert a continuous surface representing relative climatic suitability into a binary map that displays suitable and unsuitable regions. A variety of thresholding criteria have been developed for this purpose [37], [38]. We converted the continuous Maxent outputs of relative climatic suitability into binary grid files using two threshold criteria: (1) the generous minimum training presence (MTP) threshold, sometimes termed ‘lowest presence threshold;’ and (2) the more stringent maximum training sensitivity plus specificity (MTSS) threshold. The MTP threshold reduces errors of omission; cells were coded “suitable” if the Maxent output suitability value was greater than or equal to the lowest output value for the training occurrence points on any of the ten runs for a given species and time period (modern or historical). The MTSS threshold represents the Maxent output suitability value that maximizes the sum of sensitivity and specificity obtained from the error matrix [73] for the training data. The MTSS threshold balances errors of omission and commission and has found a high degree of support when evaluated against other thresholding methods across a range of prevalence values [37], [38]. Cells with Maxent output values greater than or equal to the MTSS threshold for any of the ten runs for a given species and time period (modern or historical) were coded as “suitable.” We displayed the average logistic output values for Maxent for each set of 10 runs, which can be interpreted as an index of relative climatic suitability scaled from 0–1, the cumulative MTP threshold (any cell with at least one run above the MTP threshold  = 1, otherwise  = 0) and the cumulative MTSS threshold (any cell with at least one run above the MTSS threshold  = 1, otherwise  = 0) on each map to assist with comparisons. All maps were produced using ArcMap 9.3.1 [51].

We evaluated the performance of the MTP and MTSS thresholds using independently generated presence/absence test data (see Text S5) and the Kappa statistic, or the proportion of specific agreement [73]. The thresholded Maxent model outputs generated using pseudo-presence points from the modern range were evaluated using test files generated from the modern range. Likewise, thresholded model outputs generated from the historical range were evaluated using test files generated from the historical range. We also evaluated model outputs generated from the modern range using test files generated from the historical range to see how well training data from contracted modern distributions could predict historical distributions.

## Results

### Modeling Native Ranges

Maxent performed well at interpolating climatic suitability for modern and historical time periods in native ranges (Table 3 and Figures S3-S6). For models using pseudo-presence training points generated within the modern range, Maxent predictions of climatic suitability had high correspondence with pseudo-presence testing points from the modern range for all focal species (mean AUC values >0.91) and there was low variation in AUC scores across the ten runs using different sets of pseudo-presence points (Table 3). Similarly, for models using pseudo-presence training points generated within the historical range, Maxent predictions of climatic suitability had high correspondence with testing points from the historical range (mean AUC values >0.80) with low variation between runs (Table 3). MTSS thresholds outperformed MTP thresholds across all species and time periods when there was temporal correspondence between the training and testing data (Table 3). By contrast, when models generated from contracted modern ranges were used to predict more expansive historical ranges, MTSS thresholds had slightly lower performance than MTP thresholds for all species except the oryx (Table 3). For the oryx, the more stringent MTSS threshold was more accurate in all cases due to the fact that the oryx's modern and historical ranges were fairly similar.

**Table 3:** Performance of Maxent models in predicting climatic suitability in modern (m) or historical (h) native ranges.

Species	AUC (mean ± SD)	Kappa_MTP_	Kappa_MTSS_	Kappa_MTP_ *	Kappa_MTSS_ *
Asian elephant (m)	0.976±0.003	0.703	0.768	0.385	0.320
Asian elephant (h)	0.935±0.007	0.499	0.703	-	-
Cheetah (m)	0.913±0.013	0.425	0.661	0.658	0.554
Cheetah (h)	0.805±0.016	0.581	0.797	-	-
Lion (m)	0.944±0.004	0.512	0.690	0.403	0.376
Lion (h)	0.865±0.011	0.410	0.600	-	-
Oryx (m)	0.961±0.005	0.465	0.780	0.543	0.779
Oryx (h)	0.953±0.006	0.502	0.770	-	-

Note: Models were tested using random pseudo-presence data that was generated separately from training data. The AUC values were averaged over 10 runs for each species/time period. Kappa statistics were calculated from cumulative MTP and MTSS thresholded model outputs and a set of separately generated random pseudo-presence and pseudo-absence points.

*Thesholded Maxent predictions generated using modern range training data were evaluated using test files that corresponded with historical ranges.

Temperature-associated variables made the largest contributions to the cheetah, lion and oryx models, while precipitation variables made the largest contributions to the Asian elephant models (Tables 4 and 5). Jackknife tests of single variables generally confirmed the rankings of the variable contribution values, although a few variables were much more effective at predicting testing data alone than indicated by models built using all variables (e.g., maximum temperature of the warmest month for the cheetah models based on historical pseudo-presence data and temperature seasonality for the cheetah models based on modern data). Interestingly, there were within-species differences in variable importance for models that used modern vs. historical pseudo-presence data. For example, the cheetah and lion models based on modern pseudo-presence data were affected the most by isothermality, where suitability was highest at intermediate values, while the cheetah and lion models based on historical pseudo-presence data were affected the most by annual mean temperature, which had a positive association with suitability (Table 4). The Asian elephant models based on modern pseudo-presence data were affected the most by annual precipitation, which had a positive association with suitability, while models based on historical pseudo-presence data were affected the most by precipitation of the wettest quarter, which had a also had a positive association with suitability (Table 5). For the oryx, isothermality contributed the most to models based on both modern and historical data, where suitability was highest at intermediate values, followed by precipitation of the wettest quarter, which had a negative association with suitability in both time periods (Tables 4 and 5).

**Table 4:** Percent contribution (mean ± SD) of six temperature-associated bioclimatic variables^1^ to Maxent models of climatic suitability.

Bioclimatic Variable:	MTEMP	TEMPR	ISO	TEMPS	MTWM	MTCM
Asian elephant (m)	1.6±1.6	5.6±2.7	11.5±2.2	2.1±3.2	0.8±0.7	4.3±5.7
Asian elephant (h)	30.2±6.5	4.4±2.9	16±3.0	2.2±1.0	4.6±2.4	3±4.7
Cheetah (m)	11.6±9.8	14.6±4.9	**42.4±10.4**	5.3±2.8	4.4±2.6	1.9±1.1
Cheetah (h)	**32.9±14.1**	15.9±9.3	9.7±12.8	2.5±1.1	8.6±8.8	12.2±9.3
Lion (m)	5.2±2.6	3.4±2.8	**62.5±8.7**	8.8±9.8	1.0±0.8	0.7±0.9
Lion (h)	**23.9±12.3**	6.2±3.4	23.3±13.3	8.3±4.7	6.4±4.7	13.7±16.3
Oryx (m)	1.5±1.3	4.3±1.9	**43.6±2.8**	2.5±1.4	8.1±4.3	2±1.6
Oryx (h)	2±1.6	2.9±1.5	**47.5±2.6**	2.7±1.1	8.1±2.7	1.1±0.8
Average	13.6±6.2	7.2±3.7	32.1±7.0	4.3±3.15	5.3±3.4	4.9±5.1

Note: Variable contributions were averaged over ten model runs for each species and time period. The variables with the largest contribution for each species and time period are shown in bold; m  =  models trained with pseudo-presence data from the modern range; h  =  models trained with pseudo-presence data from the historical range.

1 MTEMP  =  Annual mean temperature; TEMPR =  Mean monthly temperature range; ISO  =  Isothermality (mean monthly temperature range/temperature annual range); TEMPS  =  Temperature seasonality (standard deviation of monthly temperature); MTWM  =  Maximum temperature of the warmest month; and MTCM  =  Minimum temperature of the coldest month.

**Table 5:** Percent contribution (mean ± SD) of four precipitation-associated bioclimatic variables^2^ to Maxent models of climatic suitability.

Bioclimatic Variable:	PREC	PRECS	PWQ	PDQ
Asian elephant (m)	**35.1±8.1**	1.5±1.3	28.7±11.7	8.8±1.9
Asian elephant (h)	2.3±2.2	3.7±1.7	**30.8±6.1**	2.8±2.6
Cheetah (m)	6.4±2.7	6.0±4.8	1.8±1.3	5.6±4.6
Cheetah (h)	1.9±1.4	2.1±1.0	1.7±1.4	12.3±8.8
Lion (m)	8.0±2.3	6.9±1.8	1.3±0.9	2.1±1.7
Lion (h)	4.5±2.2	6.0±4.6	3.5±2.9	4.1±2.0
Oryx (m)	6.9±3.6	8±4.5	22.1±3.4	1±0.6
Oryx (h)	11.2±4	7.1±3.6	15.9±3.5	1.5±1.3
Average	9.5±3.3	5.2±2.9	13.2±3.9	4.8±2.9

Note: Variable contributions were averaged over ten model runs for each species and time period. The variables with the largest contribution for each species and time period are shown in bold; m  =  models trained with pseudo-presence data from the modern range; h  =  models trained with pseudo-presence data from the historical range.

2 PREC  =  Annual precipitation; PRECS  =  Precipitation seasonality (coefficient of variation of monthly precipitation); PWQ  =  Precipitation of the wettest quarter; and PDQ  =  Precipitation of the driest quarter.

### Model Projections in North America

**Figure 1:**
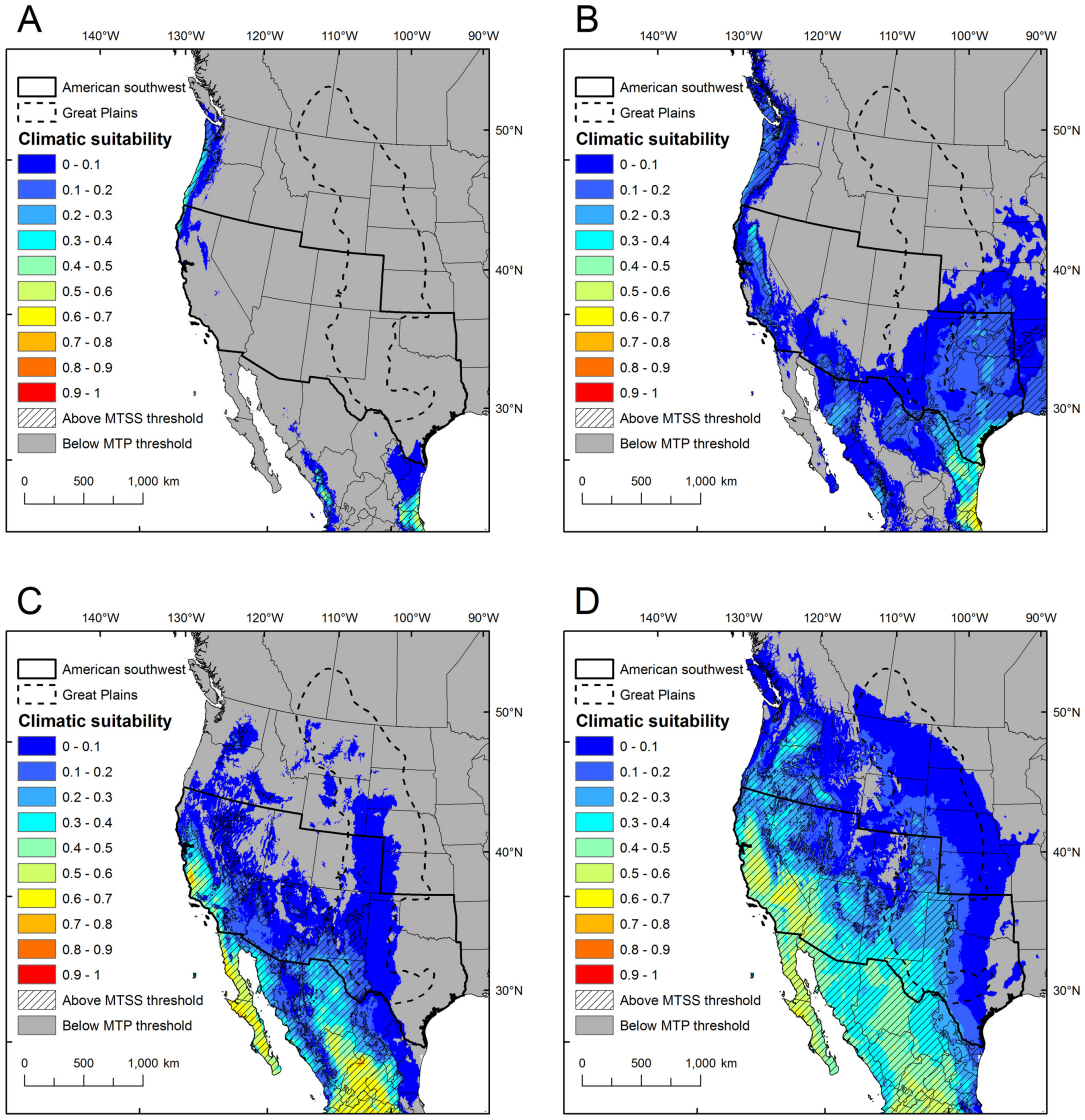
Predicted climatic suitability for the Asian elephant and cheetah in North America. Climatic suitability for the Asian elephant is based on pseudo-presence points from the modern (A) and historical (B) range, and for the cheetah on pseudo-presence points from the modern (C) and historical (D) range. “Climatic suitability” is the average of ten Maxent logistic outputs per species per time period, where blue indicates low suitability and red indicates high suitability. Regions above the MTSS threshold are shown as hashed areas, while regions below the MTP threshold are shown in gray. The proposed introduction areas under the Pleistocene rewilding proposal (the Great Plains and American southwest) are outlined.

**Figure 2:**
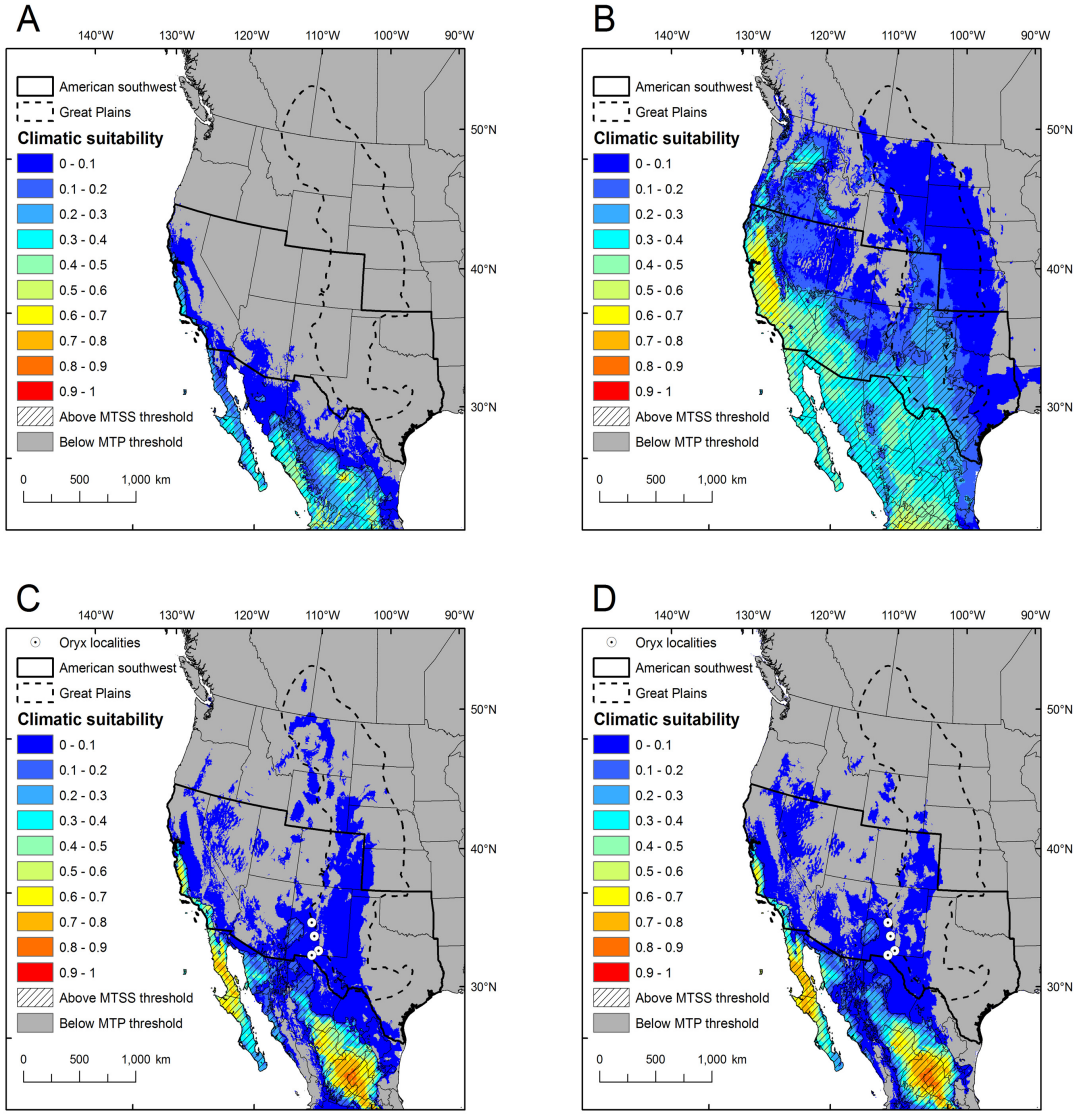
Predicted climatic suitability for the lion and oryx in North America. Climatic suitability for the lion is based on pseudo-presence points from the modern (A) and historical (B) range, and for the oryx on pseudo-presence points from the modern (C) and historical (D) range. Four localities where oryx have established wild populations are shown as white circles. “Climatic suitability” is the average of ten Maxent logistic outputs per species per time period, where blue indicates low suitability and red indicates high suitability. Regions above the MTSS threshold are shown as hashed areas, while regions below the MTP threshold are shown in gray. The proposed introduction areas under the Pleistocene rewilding proposal (the Great Plains and American southwest) are outlined.

Projections in North America from modern pseudo-presence training points generally indicated low climatic suitability for the Asian elephant and lion in the Great Plains, while the cheetah projections had some areas above the MTSS threshold in Texas and New Mexico (Figures 1A, 1C, 2A and 2C). Similarly low climatic suitability was found in the American southwest for the Asian elephant and lion, except for coastal California that had some areas above the MTSS threshold (Figures 1A and 2A). The cheetah had more extensive areas above the MTSS threshold in Arizona, California, Nevada, New Mexico and Texas (Figure 1C). For the oryx, the most suitable areas above the MTSS threshold were in restricted regions in coastal California and in a small region of the American southwest in New Mexico and Arizona, while portions of the Great Plains were above the MTP threshold (Figure 2C). All four localities where oryx have established in North America were above the MTP threshold, but none were above the MTSS threshold (Figure 2C).

By contrast, projections in North America from historical pseudo-presence training points showed higher levels and more extensive areas of climatic suitability in the Great Plains and American southwest than projections from modern range training data for the Asian elephant, cheetah and lion (Figures 1B, 1D and 2B), while the oryx projections were similar but showed a slightly smaller climatically suitable area (Figure 2D). The Asian elephant projections indicated low to medium climatic suitability across a wider region along the West coast and in portions of the American southwest and the southern Great Plains (Figure 1B). The cheetah and lion projections indicated medium and/or high climatic suitability in portions of Oregon, Washington, Idaho, California, Nevada, Arizona, New Mexico and Texas, with low climatic suitability extending over most of the remaining Great Plains and American southwest (Figures 1D and 2B). Again, all four localities where oryx have established in North America were above the MTP threshold, but none were above the MTSS threshold (Figure 2D).

## Discussion

The use of modern vs. historical training data had a substantial effect on model predictions for all species except the oryx. Larger zones of potential climatic suitability were predicted in North America from models fit to historical training data compared to those fit to modern training data for the Asian elephant, cheetah and lion (Figures 1A, 1B, 1C, 1D, 2A and 2B). This result is explained by the fact that all three species have contracted modern ranges characterized by a loss of area in colder northern latitudes (Figures S3, S4 and S5). In the native range, models fit to modern training data for the same three species performed poorly when evaluated with historical testing data (Table 3). The inability to predict past distributions using training data from modern, contracted distributions is consistent with previous findings showing that the degree of sampling bias with respect to climatic conditions has a negative effect on predictive accuracy [75]. By contrast, for the oryx there was little difference in the predicted zones of climatic suitability in North America between models fit to modern vs. historical training data (Figures 2C and 2D), resulting from the fact that its modern range was only slightly reduced from its historical range (Figure S6). There was also no difference in the ability of models fit to modern vs. historical training data to predict the four North American oryx localities; both model groups successfully predicted the localities at the generous MTP threshold but failed to predict them with the more stringent MTSS threshold. These results suggest that the use of historical occurrence data for model training can improve performance, at least in the native range, but the magnitude of this effect is dependent on the degree to which modern and historical ranges for each species differ.

The threshold criterion (MTP or MTSS) had a substantial effect on model predictions for all species. The MTSS threshold outperformed the MTP threshold for all focal species in the native range (Table 3) and is recognized as one of the better-performing threshold criteria [37], [38]. However, none of the four North American oryx localities were correctly predicted using MTSS, while all four localities were correctly predicted when the lower MTP threshold was applied using both modern and historical training data (Figures 2C and 2D). This finding is reminiscent of Peterson et al. [29], who found that Maxent models performed poorly and exhibited overfitting (when a statistical model describes random error or noise instead of the underlying relationship) when used to project to unsampled regions at higher thresholds, but successfully reconstructed distributions of species at lower thresholds. Our results suggest that threshold criteria perform differently when used for interpolation in the native range than when used for extrapolation to new geographic areas; specifically, lower thresholds may be more accurate that higher ones when Maxent is used for extrapolation. Increasing the regularization multiplier may also improve Maxent's generalizability [76]. The default regularization multiplier value of 1 yielded the highest model performance for the oryx in the native range using modern training data, while a value of 0.75 yielded the highest model performance in the native range using historical training data (Figure S2). Comprehensive guidelines for how to select appropriate thresholds and regularization multipliers when extrapolating to new regions have yet to be developed.

**Figure 3:**
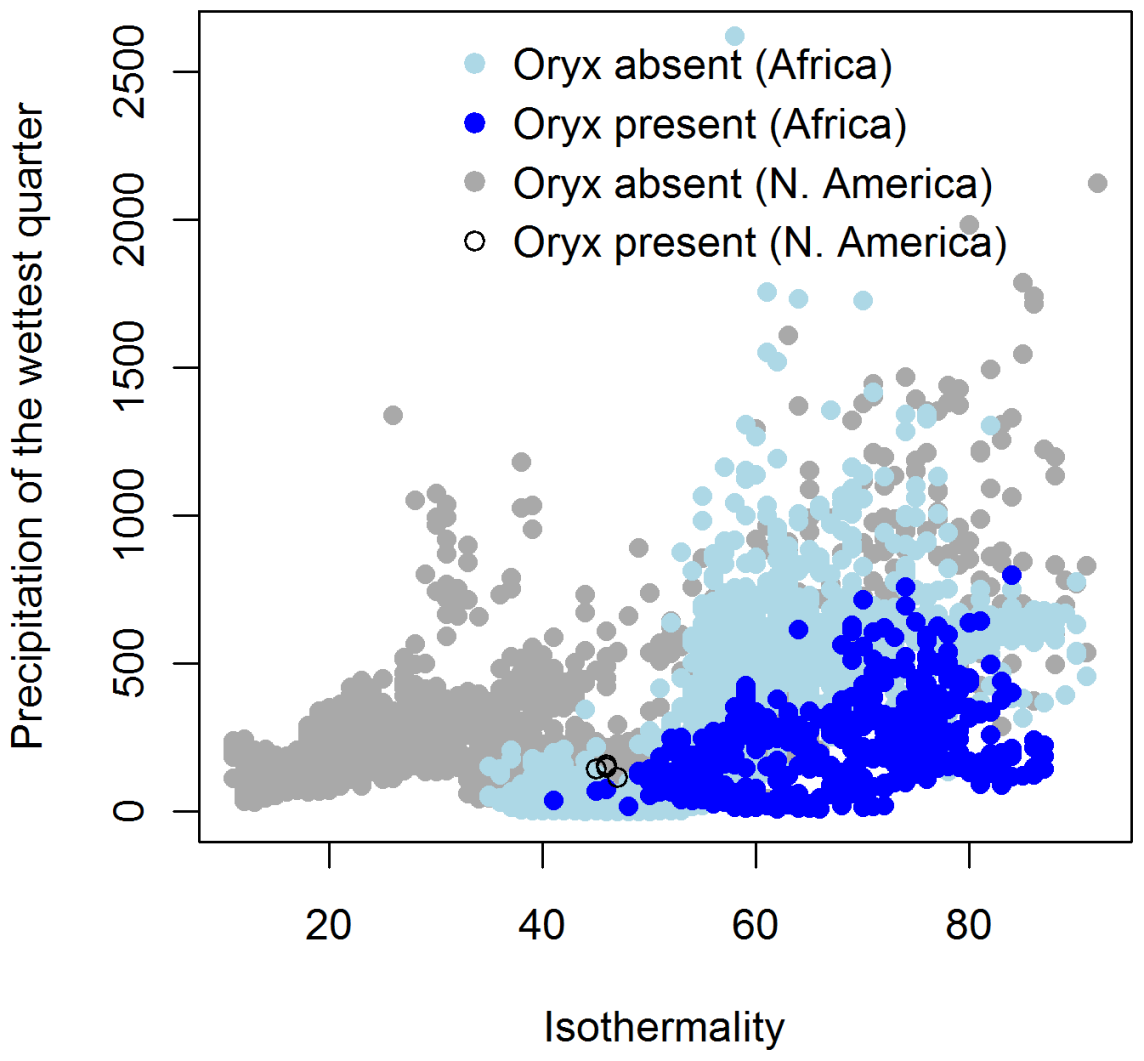
Plot of isothermality vs. precipitation in the wettest quarter for regions in Africa and North America with and without *Oryx gazella*. Isothermality is plotted against the precipitation of the wettest quarter for random points sampled within the native modern oryx range, for random points sampled across Africa and North America and for the four localities where oryx are established in North America.

Another possibility that might explain the relatively low predicted climatic suitability for the North American oryx localities could be novel combinations of climatic conditions that are suitable for the oryx in North America but that do not occur in Africa. We explored this possibility by plotting isothermality and precipitation of the wettest quarter, the two variables that made the largest contributions to the oryx models—totaling 64.6% (Tables 4 and 5), for randomly sampled points within the native modern oryx range, for random points sampled across Africa and North America and for the four localities where oryx have established in North America (Figure 3). For these two climatic variables, it appears that the North American oryx localities are indeed at the edge of the oryx climatic envelope, suggesting that the oryx may be encountering regions with novel climates in North America that have no analog in Africa. The issue of “non-analog climates” presents a special challenge for species distribution modeling across space and time and will become more problematic with climate change [22], [77]. It is difficult to test predictions of climatic suitability for the oryx in North America since it is recently established and has almost certainly not reached the limits of its potential distribution. Future research should focus on species that have invaded, established and spread throughout new geographical areas with known non-analog climates.

Maxent performed well at interpolating modern and historical distributions in native ranges for all species (Table 3). This is consistent with previous findings that have demonstrated that Maxent successfully predicts species' native distributions using occurrence data from within the same region [35], [41], [70]. The low variation in AUC values between model runs that used different random pseudo-presence data suggests that the points adequately sampled the available environmental gradients for these species; however, further research should determine the optimal amount of sampling needed at different geographical scales relative to the scale of the species occurrence data [78].

Like all species distribution modeling exercises, our results are correlative and there were inherent sources of bias at each step of the modeling process. Sources of error include the uneven distribution of climate stations that were the source of the WorldClim climate data, imperfect modern and historical range maps, the temporal mismatch between the historical range maps and the climate data and numerous decisions made during model development and implementation (see Text S1-S5, Figures S1 and S2). Since our predictions related to continental-scale distributions, we only used climatic explanatory variables, which are thought to be the main determinants of species' distributions at these very large scales [79]. The climatic variables that we used as predictors were for the most part only proximate factors, not direct (physiological) factors. Our results should be interpreted, therefore, only in the context of broad-scale climatic suitability. Future analyses of potential focal species distributions at finer spatial scales, e.g., to assess the suitability of introduction sites or to delineate protected areas, would greatly benefit from incorporating landscape- or regional-scale factors such as land use, topography, geology, vegetation type, available prey populations and human population density. Land use datasets, digital elevation models, satellite imagery, soil maps, etc. are now available in digital format and could be incorporated into a GIS model for this purpose. Additional ecological effects, e.g., trophic cascades, as well as societal/ethical considerations, such as wildlife-human conflict and the risk of colonizing populations introducing infectious diseases, a serious hazard to both the original host and other spillover species [80], just to name a few issues, would need to be considered as part of a comprehensive assessment for any proposed introductions. These considerations would likely greatly reduce the potential geographical scope of introductions for rewilding species within areas that appear climatically suitable.

Proposals for introducing Asian elephants, cheetahs and lions to the American southwest and Great Plains should take climate into consideration. The importance of temperature in the modeling results, particularly annual mean temperature and isothermality, suggests that North America's overall cooler and more seasonal climate compared to Africa and southern Asia would place limits on the successful establishment of these focal species. From our results, most of the American southwest and Great Plains had low suitability for the Asian elephant, with some moderately suitable areas indicated in California, the Pacific Northwest, Texas and Oklahoma (Figures 1A and 1B). For the cheetah and lion, more extensive regions of the American southwest and southern Great Plains appeared to be suitable based purely on climatic factors, especially from models fit to historical training data (Figures 1C, 1D, 2A, 2B). If the MTP threshold has higher performance than the MTSS threshold in model projections to North America, as suggested by the oryx results, then much larger regions of the western United States may indeed be climatically suitable for cheetahs and lions than the Maxent logistic output values of relative climatic suitability indicate. The expansion of the tropical belt with climate change [81] could further increase the suitability of some regions in North America for these focal species over time.

SDMs are increasingly used to predict climatic suitability in novel geographic or temporal scenarios and require improvements in performance. Here we incorporated the use of modern and historical range information and pseudo-presence data to enhance predictions of climatic suitability across continents. Our predictions based on modern vs. historical range information led to substantially different projections of climatic suitability in three out of four focal species. Applications of SDMs that currently use only occurrence data from the modern range may be improved by incorporating historical information, when available, to account for range contractions due to non-climatic factors such as human disturbance. While species locality data is increasingly available online for some taxa, particularly mammals and birds (e.g., Manis and Ornis databases, respectively), this locational data may still be biased and/or sparse. The use of random pseudo-presence points generated from range maps is an economical approach that can address the problem of biased or incomplete sampling. This approach may be particularly useful for widespread generalist species with well-defined ranges but few museum records, especially as many SDMs are sensitive to small sample sizes [26], [82]. Pseudo-presence data are not, however, a substitute for having accurately georeferenced museum specimens, especially for narrow-ranging species and ecological specialists with narrow niche breadth. Species distribution modeling will continue to play an important role in adaptive management and conservation planning as complex challenges, such as predicting range shifts of organisms in response to climate change, are addressed. The method that we present here aimed to provide both generous and conservative predictions of climatic suitability. Our most generous predictions minimized errors of omission by using historical range information, randomly sampled pseudo-presence data and a generous threshold criterion (MTP). Our most conservative predictions minimized errors of commission by using modern range information and a more conservative threshold criterion (MTSS). The balanced nature of our procedure makes it a useful model for other applications of SDMs in ecology, evolution and conservation biology where the goal is to assess potential climatic suitability in new geographic regions or times.

## Supporting Information

Text S1 Determining the number of random pseudo-presence points.
(0.03 MB DOC)

Text S2 Geographical extent of climate grids.
(0.03 MB DOC)

Text S3 Additional information on Maxent.
(0.03 MB DOC)

Text S4 Determining the regularization multiplier.
(0.03 MB DOC)

Text S5 Model evaluation.
(0.03 MB DOC)

Figure S1 The effect of the number of random pseudo-presence points on Maxent model performance. Model performance measured as average AUC; m  =  modeled with modern range data; h  =  modeled with historical range data.
(6.08 MB TIF)

Figure S2 The effect of regularization on Maxent model performance. Model performance measured as average AUC; m  =  modeled with modern range data; h  =  modeled with historical range data.
(6.08 MB TIF)

Figure S3 Modeled climatic suitability for the Asian elephant in the native range. Climatic suitability based on pseudo-presence points from the modern (A) and historical (B) range. “Climatic suitability” is the average of ten Maxent logistic outputs per time period, where blue indicates low suitability and red indicates high suitability. Regions above the MTSS threshold are shown as hashed areas, while regions below the MTP threshold are shown in gray.
(3.07 MB TIF)

Figure S4 Modeled climatic suitability for the cheetah in the native range. Climatic suitability based on pseudo-presence points from the modern (A) and historical (B) range. “Climatic suitability” is the average of ten Maxent logistic outputs per time period, where blue indicates low suitability and red indicates high suitability. Regions above the MTSS threshold are shown as hashed areas, while regions below the MTP threshold are shown in gray.
(3.63 MB TIF)

Figure S5 Modeled climatic suitability for the lion in the native range. Climatic suitability based on pseudo-presence points from the modern (A) and historical (B) range. “Climatic suitability” is the average of ten Maxent logistic outputs per time period, where blue indicates low suitability and red indicates high suitability. Regions above the MTSS threshold are shown as hashed areas, while regions below the MTP threshold are shown in gray.
(3.35 MB TIF)

Figure S6 Modeled climatic suitability for *Oryx gazella* in the native range. Climatic suitability based on pseudo-presence points from the modern (A) and historical (B) range. “Climatic suitability” is the average of ten Maxent logistic outputs per time period, where blue indicates low suitability and red indicates high suitability. Regions above the MTSS threshold are shown as hashed areas, while regions below the MTP threshold are shown in gray.
(2.38 MB TIF)
