# Supplementary material for: Is the Climate Right for Pleistocene Rewilding? Using Species Distribution Models to Extrapolate Climatic Suitability for Mammals across Continents
Source: PLoS One. 2010 Sep 22;5(9):e12899. doi: 10.1371/journal.pone.0012899 (PMC2943917; doi:10.1371/journal.pone.0012899)
Supplement: Text S1 — Determining the number of random pseudo-presence points. (0.03 MB DOC) [file pone.0012899.s001.doc]

To determine the number of random pseudo-presence points to be used, we compared Maxent model performance using ten sets each of 10, 50, 100, 150, and 200 points for the four focal species in two time periods (modern and historical). We found that model performance was near maximal with 100 pseudo-presence points across species and time periods (Figure S1). This result is comparable with previous studies that have examined the effect of sample size on model performance [1-3].

References

1. Wisz MS, Hijmans RJ, Li J, Peterson AT, Graham CH, et al. (2008) Effects of sample size on the performance of species distribution models. Divers Distrib 14: 763-773.

2. Stockwell DRB, Peterson AT (2002) Effects of sample size on accuracy of species distribution models. Ecol Model 148: 1-13.

3. Hernandez PA, Graham CH, Master LL, Albert DL (2006) The effect of sample size and species characteristics on performance of different species distribution modeling methods. Ecography 29: 773-785.
