# Supplementary material for: Is the Climate Right for Pleistocene Rewilding? Using Species Distribution Models to Extrapolate Climatic Suitability for Mammals across Continents
Source: PLoS One. 2010 Sep 22;5(9):e12899. doi: 10.1371/journal.pone.0012899 (PMC2943917; doi:10.1371/journal.pone.0012899)
Supplement: Text S2 — Geographical extent of climate grids. (0.03 MB DOC) [file pone.0012899.s002.doc]

The spatial extent of the environmental layers relative to the extent of species presence data can have impacts on model performance and variable importance [1]. For native range modeling we clipped the worldwide climate grid files to include all of Africa, Europe, and most of Asia (eastern boundary at approximately 138° E) for all model runs. For projections to North America, we clipped the worldwide climate grid files to include all of North America, Central America, and part of South America (southern boundary at approximately 4° N). Thus, the same climate grids were used regardless of species or time period.

Reference

1. VanDerWal J, Shoo LP, Graham C, William SE (2009) Selecting pseudo-absence data for presence-only distribution modeling: How far should you stray from what you know? Ecol Model 220: 589-594.
