# Supplementary material for: Is the Climate Right for Pleistocene Rewilding? Using Species Distribution Models to Extrapolate Climatic Suitability for Mammals across Continents
Source: PLoS One. 2010 Sep 22;5(9):e12899. doi: 10.1371/journal.pone.0012899 (PMC2943917; doi:10.1371/journal.pone.0012899)
Supplement: Text S3 — Additional information on Maxent. (0.03 MB DOC) [file pone.0012899.s003.doc]

Maxent provides for flexibility through a number of model settings that can be adjusted by the user. We allowed the use of all feature types (linear, quadratic, product, threshold, and discrete) to constrain the computed probability distribution. Maxent determines the relative importance of each variable in describing species’ distributions using a jackknife procedure. We selected the logistic output, which can be interpreted as relative climatic suitability, because it was shown to achieve the best performance relative to cumulative and raw outputs [1]. We used default values for all other settings (maximum iterations, number of background points, convergence threshold, etc.).

For the projection of climatic suitability into North America, the parameter values computed for the native range were used together with environmental layers for North America [2]. Projecting to new areas is problematic if values of the environmental layers in the projected region fall outside the range of values from the native region. Maxent utilizes a clamping procedure that holds environmental layer values in the projection environment that fall above or below the range of values in the native region to the maximum or minimum values of the native region, respectively. Feature values derived from projection layers are also clamped if they are greater than the maximum or less than the minimum than those derived from the training data. Clamping helps to alleviate problems that can arise from making predictions outside the range of climatic data used in the model from the native region (“non-analog” climates), however the results from projections should still be interpreted with caution because sequential univariate clamping may not identify multivariate combinations of non-analog conditions [3].

References

1. Phillips SJ, Dudík M (2008) Modeling of species distributions with Maxent: new extensions and a comprehensive evaluation. Ecography 31: 161-175.

2. Phillips SJ, Anderson RP, Schapire RE (2006) Maximum entropy modeling of species geographic distributions. Ecol Model 190: 231-259.

3. Fitzpatrick MC, Hargrove WW (2009) The projection of species distribution models and the problem of non-analog climate. Biodivers Conserv 18: 2255–2261.
