# Supplementary material for: Is the Climate Right for Pleistocene Rewilding? Using Species Distribution Models to Extrapolate Climatic Suitability for Mammals across Continents
Source: PLoS One. 2010 Sep 22;5(9):e12899. doi: 10.1371/journal.pone.0012899 (PMC2943917; doi:10.1371/journal.pone.0012899)
Supplement: Text S4 — Determining the regularization multiplier. (0.03 MB DOC) [file pone.0012899.s004.doc]

Regularization parameters reduce over-fitting by allowing the algorithm to maximize the entropy of the distribution subject to a relaxed constraint that feature expectations be close to feature averages over sample locations, rather than exactly equal to them [1]. We used the default regularization settings, recently described by Phillips and Dudík [2]. The regularization settings can be further adjusted by a regularization multiplier. We compared model performance using regularization multipliers of 0.05, 0.25, 0.5, 0.75, 1, 2, 5, and 10 for all species (Figure S2). A regularization multiplier of 1 (the default), yielded the highest model performance for the cheetah (modern; hereafter “m”), Asian elephant (historical; hereafter “h”), lion (h) and oryx (m); a multiplier of 2 yielded the highest performance for the lion (m) and cheetah (h); and a multiplier of 0.75 yielded the highest performance for the oryx (h). However, in these latter three cases the performance differed only by ≤0.002 AUC points from using a multiplier of 1. A multiplier of 0.5 yielded the highest performance for Asian elephant (m), which was 0.003 AUC points higher than the performance using the default of 1. We decided to use a regularization multiplier of 1 for all species and time periods, as this value yielded the highest, or nearly the highest, performance in most cases. Further studies should explore how sensitive Maxent model outputs are to adjustments to the regularization multiplier.

References

1. Phillips SJ, Anderson RP, Schapire RE (2006) Maximum entropy modeling of species geographic distributions. Ecol Model 190: 231-259.

2. Phillips SJ, Dudík M (2008) Modeling of species distributions with Maxent: new extensions and a comprehensive evaluation. Ecography 31: 161-175.
