# Supplementary material for: Is the Climate Right for Pleistocene Rewilding? Using Species Distribution Models to Extrapolate Climatic Suitability for Mammals across Continents
Source: PLoS One. 2010 Sep 22;5(9):e12899. doi: 10.1371/journal.pone.0012899 (PMC2943917; doi:10.1371/journal.pone.0012899)
Supplement: Text S5 — Model evaluation. (0.03 MB DOC) [file pone.0012899.s005.doc]

To evaluate the performance of the MTP and MTSS thresholds, we generated independent test data for each species and time period (modern and historical) in DIVA-GIS [1]. Test files consisted of 333 random pseudo-presence points generated from within the modern or historical range and 666 pseudo-absence points generated in a longitude-latitude bounding box containing the range shapefile plus an additional terrestrial buffer area 0.5 times the size of the bounding box. We used the model evaluation tools in DIVA-GIS to obtain Kappa statistics.

Reference
