# Supplementary figures and images for: Is the Climate Right for Pleistocene Rewilding? Using Species Distribution Models to Extrapolate Climatic Suitability for Mammals across Continents
Source: PLoS One. 2010 Sep 22;5(9):e12899. doi: 10.1371/journal.pone.0012899 (PMC2943917; doi:10.1371/journal.pone.0012899)

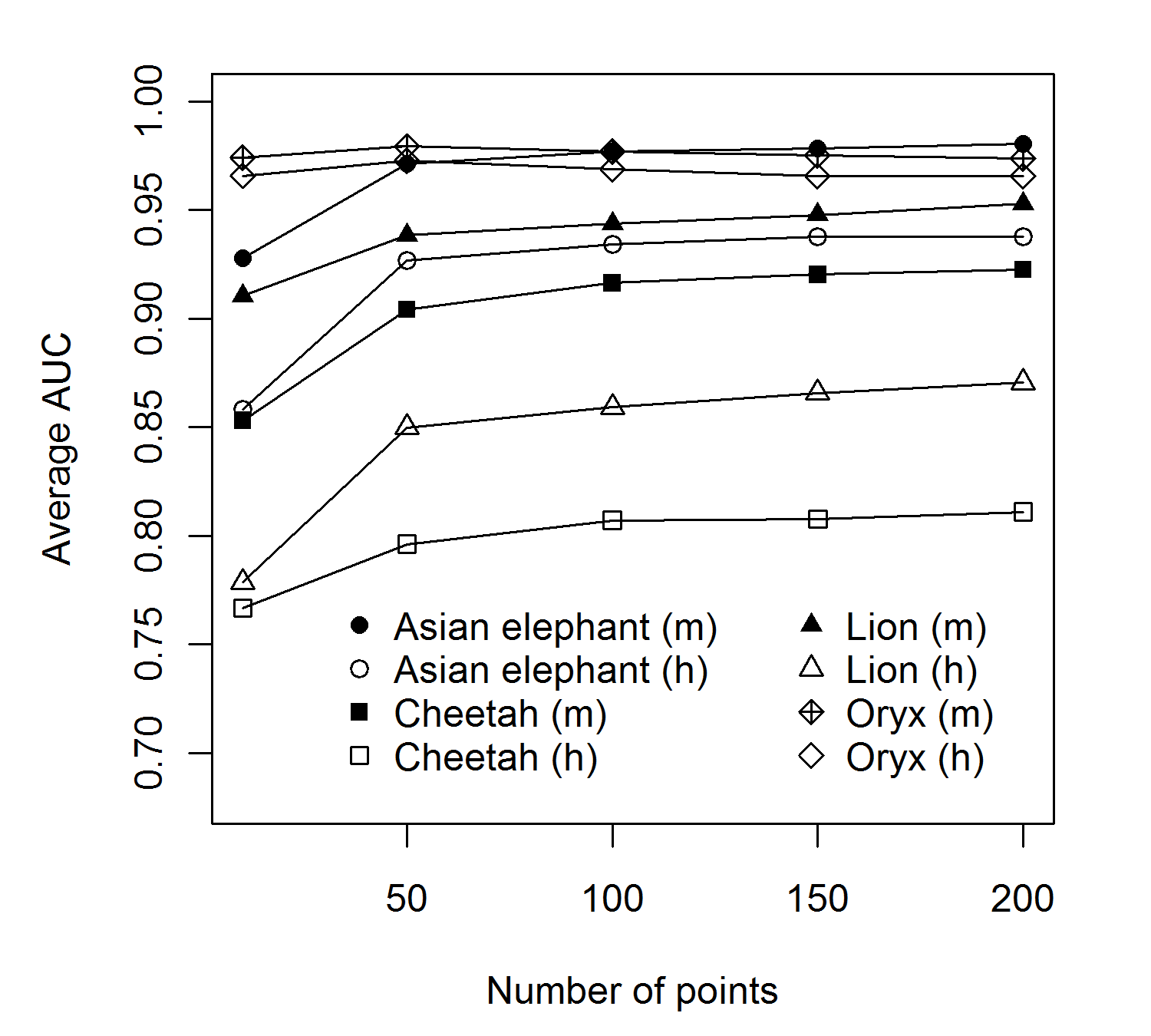

Supplement: Figure S1 — The effect of the number of random pseudo-presence points on Maxent model performance. Model performance measured as average AUC; m = modeled with modern range data; h = modeled with historical range data. (6.08 MB TIF) [file pone.0012899.s006.tif]

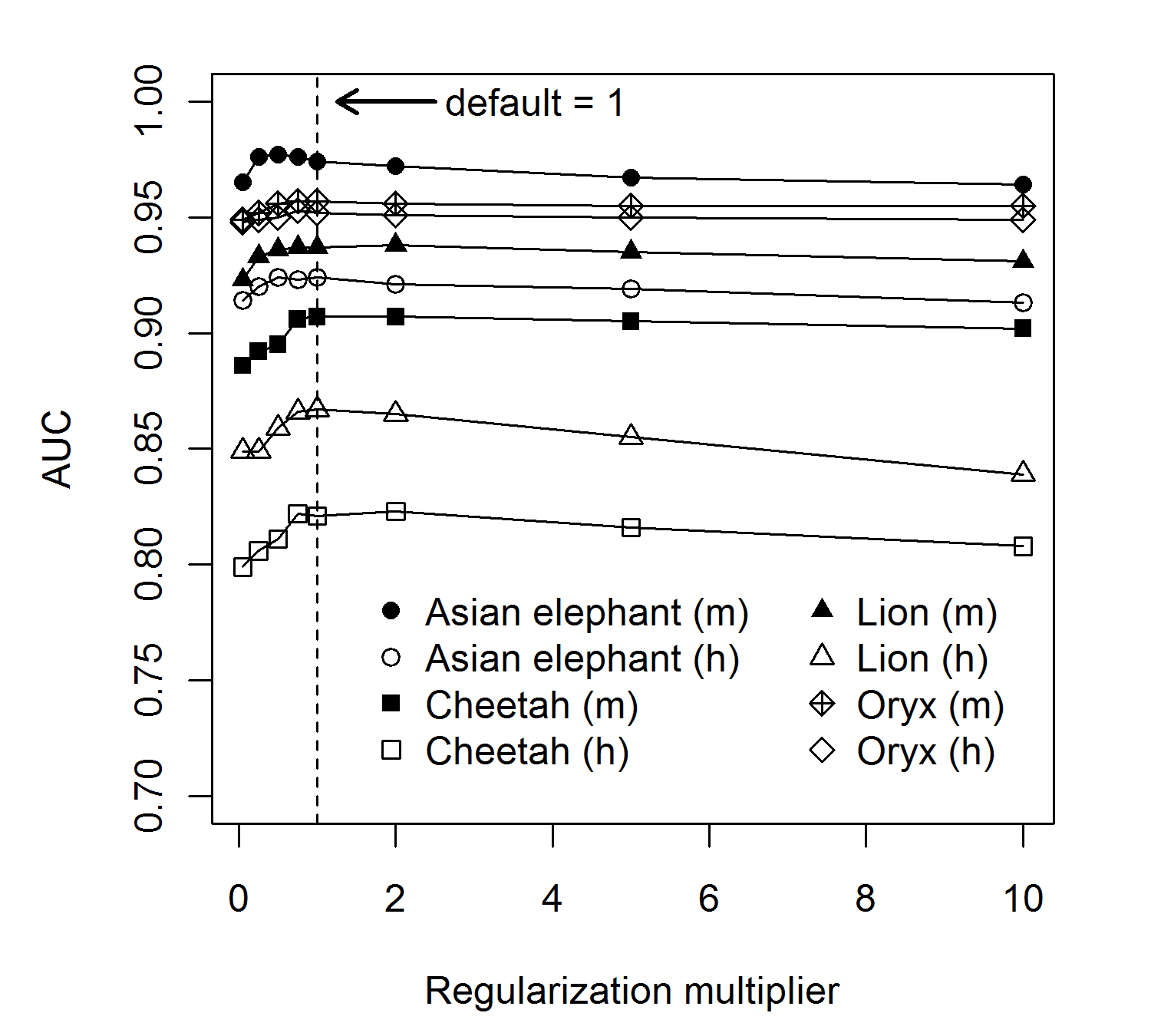

Supplement: Figure S2 — The effect of regularization on Maxent model performance. Model performance measured as average AUC; m = modeled with modern range data; h = modeled with historical range data. (6.08 MB TIF) [file pone.0012899.s007.tif]

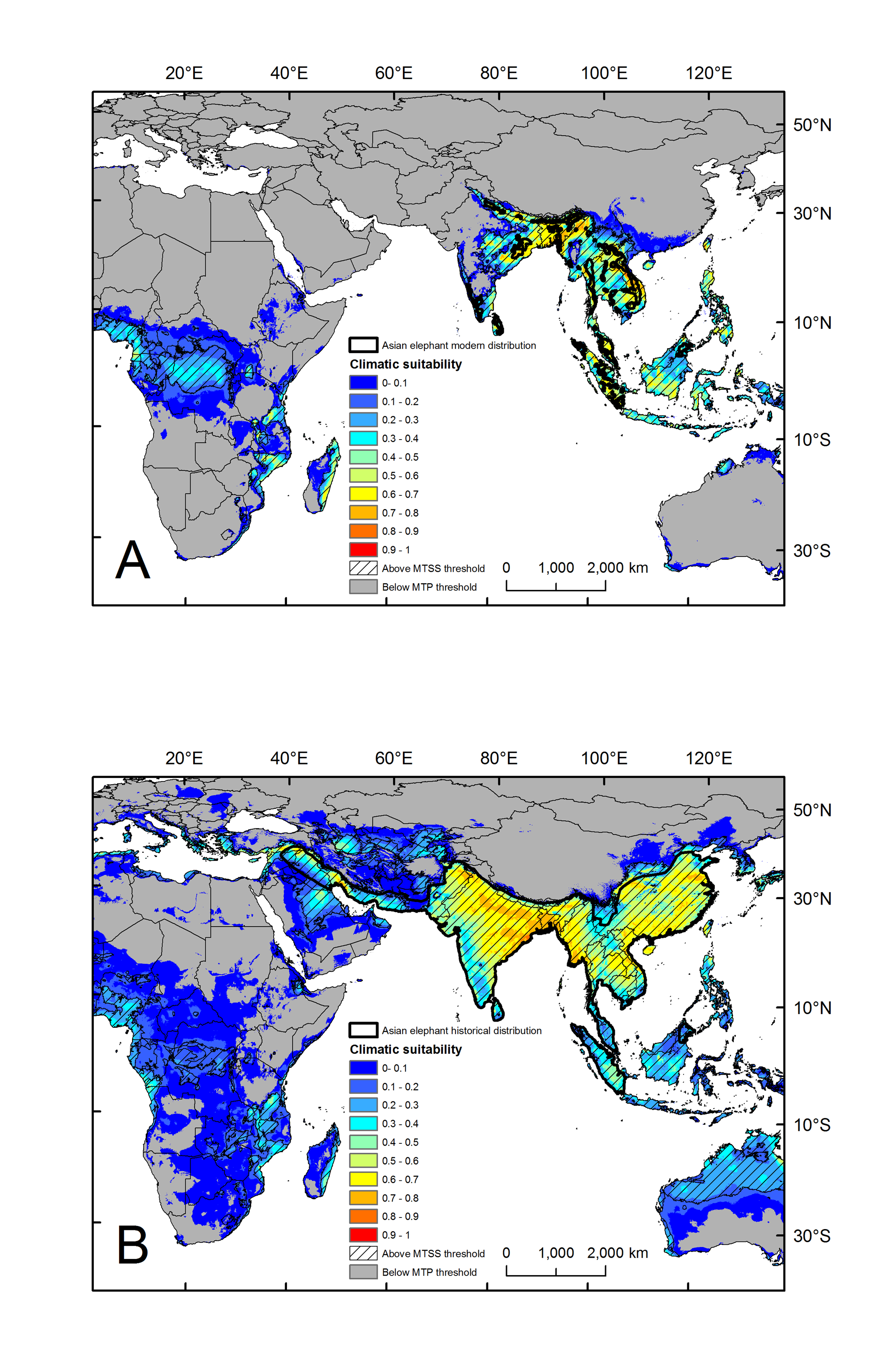

Supplement: Figure S3 — Modeled climatic suitability for the Asian elephant in the native range. Climatic suitability based on pseudo-presence points from the modern (A) and historical (B) range. “Climatic suitability” is the average of ten Maxent logistic outputs per time period, where blue indicates low suitability and red indicates high suitability. Regions above the MTSS threshold are shown as hashed areas, while regions below the MTP threshold are shown in gray. (3.07 MB TIF) [file pone.0012899.s008.tif]

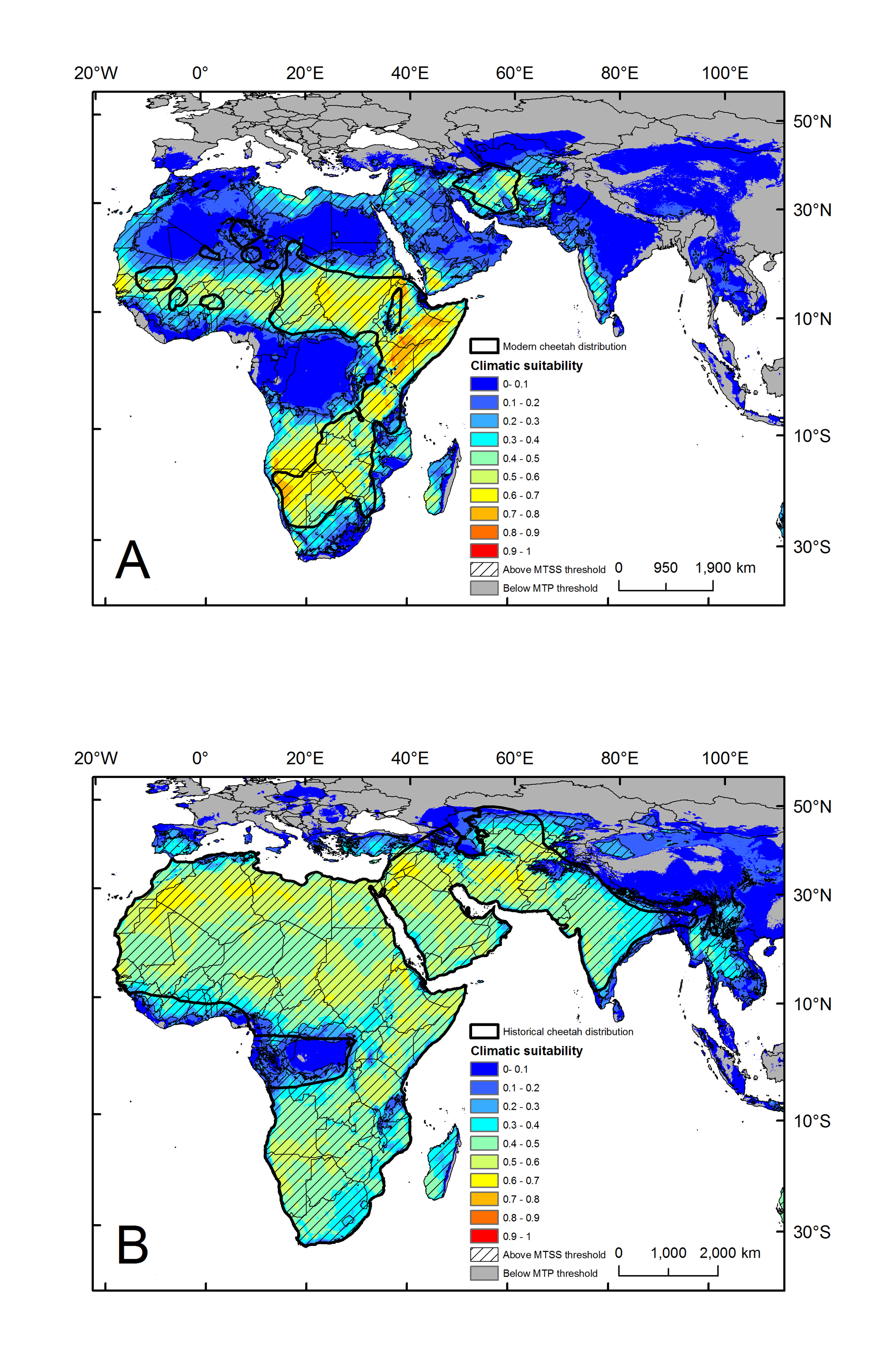

Supplement: Figure S4 — Modeled climatic suitability for the cheetah in the native range. Climatic suitability based on pseudo-presence points from the modern (A) and historical (B) range. “Climatic suitability” is the average of ten Maxent logistic outputs per time period, where blue indicates low suitability and red indicates high suitability. Regions above the MTSS threshold are shown as hashed areas, while regions below the MTP threshold are shown in gray. (3.63 MB TIF) [file pone.0012899.s009.tif]

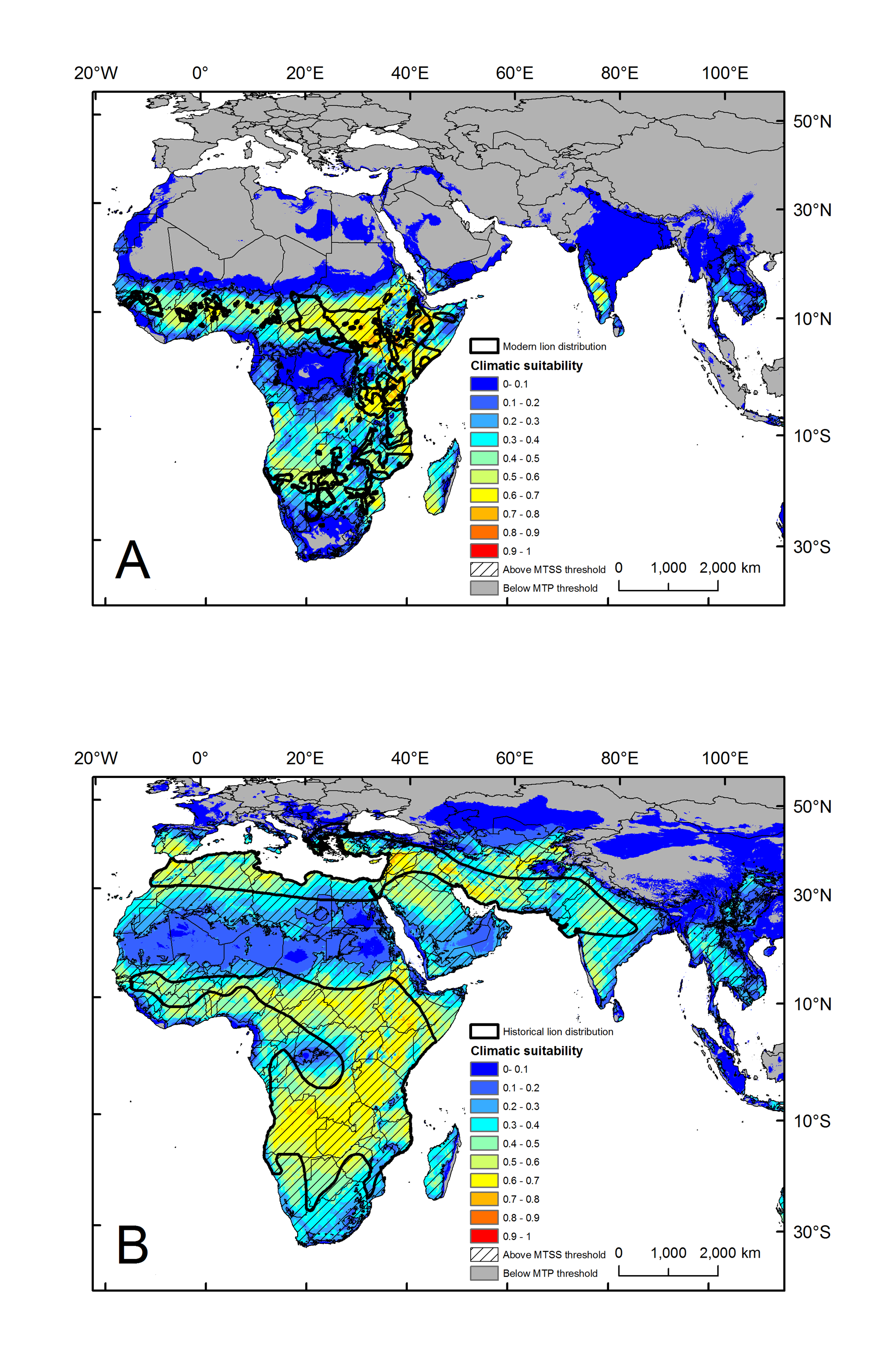

Supplement: Figure S5 — Modeled climatic suitability for the lion in the native range. Climatic suitability based on pseudo-presence points from the modern (A) and historical (B) range. “Climatic suitability” is the average of ten Maxent logistic outputs per time period, where blue indicates low suitability and red indicates high suitability. Regions above the MTSS threshold are shown as hashed areas, while regions below the MTP threshold are shown in gray. (3.35 MB TIF) [file pone.0012899.s010.tif]

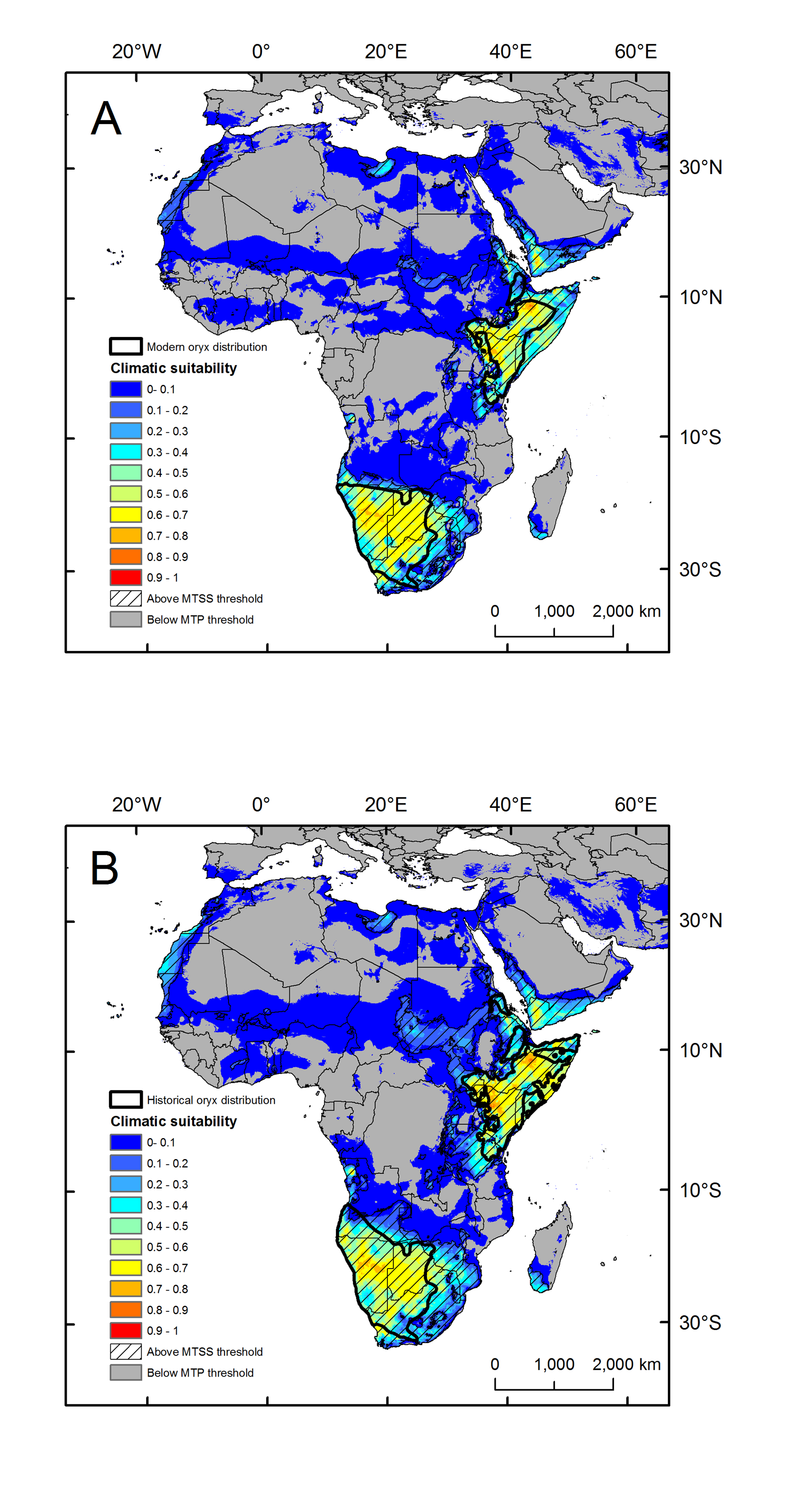

Supplement: Figure S6 — Modeled climatic suitability for Oryx gazella in the native range. Climatic suitability based on pseudo-presence points from the modern (A) and historical (B) range. “Climatic suitability” is the average of ten Maxent logistic outputs per time period, where blue indicates low suitability and red indicates high suitability. Regions above the MTSS threshold are shown as hashed areas, while regions below the MTP threshold are shown in gray. (2.38 MB TIF) [file pone.0012899.s011.tif]
